# Graves' disease treated by complementary medicine leading to thyroid storm: A case report

**DOI:** 10.22088/cjim.12.0.371

**Published:** 2021

**Authors:** Linda Daffini, Ilenia Pirola, Giovanni Saccà, Massimo Salvetti, Carlo Cappelli

**Affiliations:** 1 Department of Clinical and Experimental Sciences, SSD Medicina ad indirizzo Endocrino-Metabolico, University of Brescia, ASST Spedali Civili di Brescia, Italy; 2 Department of Clinical and Experimental Sciences; 3 University of Brescia-Spedali Civili di Brescia, Italy

## Abstract

**Background:**

Thyroid storm is a rare, life-threatening condition characterized by severe clinical manifestations of thyrotoxicosis and can be precipitated by several factors. We described a thyroid storm precipitated by a long-term treatment using homeopathic medicine containing iodine.

**Case presentation:**

A 55-year-old Italian woman was admitted to our Sub-Intensive Care Unit with the diagnosis of congestive heart failure and thyrotoxicosis. She has been diagnosed with Graves’ disease two years before; she refused conventional therapy and in the preceding six months had been using phytotherapeutic and homeopathic medicine. We found serum and urine iodine levels consistent with severe intoxication by iodine (serum iodine: 42100 mcg/L and urinary iodine: 4223 mcg/L, respectively). After a few hours, the patient went into cardiac arrest. She was subjected to invasive ventilation, dialyzed with continuous veno-venous hemofiltration and treated with vasoactive amines.

**Conclusion:**

The high level of iodemia manifested in our patient - around a thousand times greater than the normal range and itself associated with fatal outcomes - was caused by long-term homeopathic treatment. This long-term treatment has two consequences: first, iodine load-precipitated hyperthyroidism in thyroid storm, and secondly, it prevents us from treating patients with inorganic iodide.

Thyroid storm is a rare, life-threatening condition characterized by severe clinical manifestations of thyrotoxicosis (1). Although thyroid storm can develop in patients with long-standing untreated hyperthyroidism, it is often precipitated by an acute event such as thyroid or non-thyroidal surgery, trauma, infection and acute iodine load (2). Natural health products are promoted to the public as equally effective as or more effective than conventional drugs, and an increasing number of patients have resorted to them in the prevailing belief that herbal products are safe because they are natural. However, some “natural” medicines are known to have adverse effects that can sometimes be serious and even life-threatening (3). We describe a 55-year-old woman affected by Graves’ disease treated with phytotherapeutic and homeopathic medicine, which developed into life-threatening thyroid storm. 

## Case presentation

**Figure 1:**
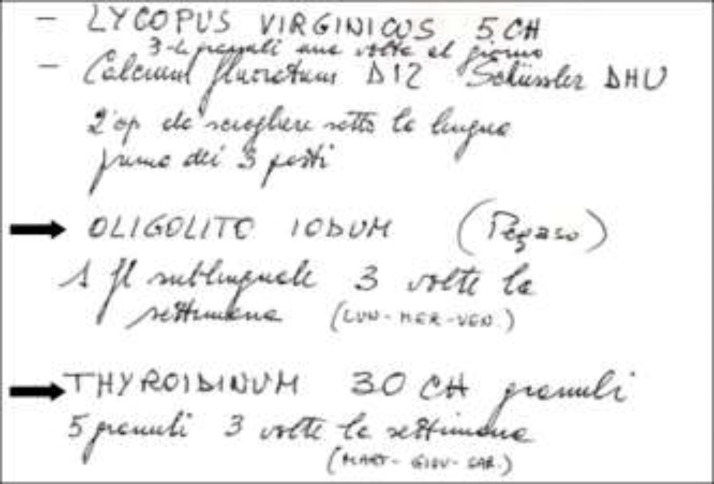
Phytotherapeutic and homeopathic prescription. Products containing iodine and extracts of sheep thyroid are marked with arrows. Oligolito Iodum: potassium iodide equal to .0024 mg of iodine; Thyroidium 30 ch: dried thyroid gland of the sheep

A 55-year-old Italian woman was referred to the Emergency Department of our hospital because of worsening dyspnea and palpitations. Her medical history was unremarkable, with the exception of Graves’ disease diagnosed two years before. She had refused conventional therapy and in the preceding six months had resorted to phytotherapeutic and homeopathic medicine (figure 1).

In particular, she had assumed Oligolito Iodium and Thyroidinum 30 CH three times a week for the past year, which contain potassium iodide and dried thyroid gland of sheep, respectively. Physical examination showed mild agitation, tachycardia (190 beats/minute), pedal edema, fine tremors in the upper limbs and fever (37.9 °C). Arterial blood pressure was 150/80 mmHg, oxygen saturation was 94%. An electrocardiogram revealed atrial fibrillation. 

The exams showed high levels of free thyroid hormones, free tetraiodine thyroxine (fT4) >60 pg/ml (normal values: 7-18), free triiodothyronine (fT3) >30 pg/ml (normal values 2.4-4.7 pg/ml), low thyroid stimulating hormone (TSH) <0.001 mU/L (normal values 0.27-4.2 mU/L), an increase in transaminases (aspartate aminotransferase (AST) 869 U/L, alanine aminotransferase (ALT) 1314 U/L (normal values 5-35 U/L)) and creatinine (1.85 mg/dl (normal values 0.5-0.9 mg/dl). The patient was admitted to our Sub-Intensive Care Unit with the diagnosis of congestive heart failure and thyrotoxicosis. 

The patient was anti-coagulated with low molecular weight heparin. She received beta-adrenergic blockade with propranolol 40 mg every 6 hours due to the detection of atrial fibrillation. We found serum and urine iodine levels, obtained upon admission to hospital, consistent with severe intoxication by iodine (ioduria 4223 mcg/L (normal values 20-200 mcg/L) and iodiemia 42100 mcg/L (normal value 40-90 mcg/L)). The patient was treated with propylthiouracil (400 mg every 6 hours) and intravenous hydrocortisone injections (100 mg every 8 hours).

After a few hours, the patient developed pronounced bradycardia (30 beats/minute), unresponsive to the intravenous atropine therapy administered to improve cardiac frequency, severe hypotension and lethargy. An echocardiogram showed severe left ventricular systolic dysfunction (ejection fraction 20-25%). While we were carrying out the echocardiogram, she went into a cardiac arrest. She was administered cardiopulmonary resuscitation and was then transferred to the Intensive Care Unit (ICU). The patient developed severe respiratory failure, with hypoxic coma and anuric renal failure. Examinations showed severe metabolic and respiratory acidosis. 

She was subjected to invasive ventilation, dialyzed with continuous veno-venous hemofiltration (CVVH) and treated with vasoactive amines. Treatment with propylthiouracil and hydrocortisone was continued, while the propranolol was discontinued because of persistent bradycardia. The clinical picture remained critical during the first few days. On the third day, iopanoic acid was administered (500 mg twice daily) to the patient. We noticed a rapid lowering of free thyroid hormone levels with normalization of free T3 on the sixth day (table 1). Her clinical condition had also slowly improved.

**Table 1:** Laboratory findings before and after treatment with Iopanoic Acid

	**Day 1** **Hospitalization **	**Day 3** **(starting Iopanoic Acid)**	**Day 6** **(after 36 hrs of Acid Iopanoic)**
TSH (0.27-4.2 mU/L)	<0.001	<0.001	<0.001
fT4 (7-18 pg/ml)	>60	>40	30.1
fT3 (2.4-4.7 pg/ml)	>30	>30	3.6

On the twenty-sixth day, the patient was discharged from Intensive care, taking propylthiouracil 50 mg every 8 hours, and transferred to a motor rehabilitation: she was alert, collaborative and normothermic. Her thyroid hormone levels and left ventricular ejection fraction were normalized. However, she had sustained permanent damage: flaccid quadriplegia, respiratory failure in non-invasive ventilation and terminal renal failure in hemodialysis. Unfortunately, no further data are available, as the patient failed to turn up for subsequent checkups. Written informed consent was obtained from the patient.

## Discussion

This case report shows the potential negative effects of phytotherapy and homeopathic medicine in the treatment of Graves' disease, due both to the development of thyroid storm and the impossibility of using iodine treatment.

Thyroid storm is a rare manifestation of extreme thyrotoxicosis requiring prompt diagnosis and treatment (4-6). Treatment options involve applying therapies used for uncomplicated hyperthyroidism using higher doses and more frequently, with additional drugs such as glucocorticoids and iodine solution. In addition, the patient must be given full support in an ICU, since the mortality rate of thyroid storm is significant (3, 4). The inhibition of new hormone production is achieved using thionamides: propylthiouracil and methimazole. Propylthiouracil has the advantage of inhibiting peripheral conversion from T4 to T3, and can be administered orally, through nasogastric tubes or rectally (1).

Another possible therapeutic approach for patients with excessive iodine overload is the administration of perchlorate. This inhibits iodide uptake in the thyroid gland by competitively binding with sodium-iodide symporter and can also discharge iodine from the thyroid gland, thereby decreasing thyroid hormone synthesis (7).

 Thyroid hormone release can be inhibited by the administration of an acute iodine load. Iodine therapy blocks the release of pre-stored tetraiodine thyroxine (T4) and triiodothyronine (T3) from the gland and decreases iodine transport and oxidation in follicular cells. The administration of pharmacologic amounts of iodine leads to the temporary inhibition of iodine organification in the thyroid gland, thereby diminishing thyroid hormone biosynthesis; a phenomenon called the Wolff-Chaikoff effect. The oral formulation of inorganic iodide includes potassium iodide-iodine (Lugol's) solution and saturated solution of potassium iodide. Dosage in thyroid storm is ten drops (8 mg iodide/iodine per drop (0.05 mL)) of Lugol's solution three times daily, or five drops (50 mg iodide/drop (0.05 mL)) of SSKI every six hours (8). Most surgeons administer Lugol’s solution to provide 30 mg or more of iodine/day for 10 days before surgery to decrease thyroid vascularity, the rate of blood flow, and intraoperative blood loss during thyroidectomy (9). However, within two to four weeks of continuous exposure to an excess of iodine, the organification and thyroid hormone biosynthesis resume, escaping the Wolff-Chaikoff effect with the resumption of normal organification of iodine and normal thyroid function (10). Very recently, Leuştean L et al. described the exacerbation of thyrotoxicosis due to misunderstanding in the preoperative preparation, with Lugol’s solution extended for a period of about 30 days (11). To the best of our knowledge, this is the first report describing a young patient affected by Graves’ disease treated with phytotherapeutic and homeopathic medicine who developed thyroid storm. The levels of serum and urine iodine found in our patient were in fact consistent with severe intoxication by iodine, caused by the long-term consumption of phytotherapeutic and homeopathic medicine (figure 1). Indeed, we cannot be sure that Oligolito and Thyroidium were the only source of iodine, even though our patient stated that she had not taken any other preparations. Furthermore, it is important to underline that the patient had impaired renal function already at the time of hospitalization and this could have contributed to the toxicity of iodine.

In any case, and in agreement with Leuştean et al. (10), the long-term intake of iodine in our patient precipitated the development of Graves’ disease into thyroid storm and prevented us from treating the patient with inorganic iodide.

The administration of oral cholecystographic agents, such as Iopanoic acid, has been reported to be useful in the treatment of hyperthyroid patients (12). The use in therapy significantly reduces biologically active iodothyronine concentrations, with the improvement of clinical symptoms and signs of thyrotoxicosis (13, 14). The significant effect of oral cholecystographic agents on serum T3 concentration is mainly the result of the competitive inhibition of types 1 and 2 5'-monodeiodinase in the liver, brain and thyroid, blocking conversion of T4 to T3, resulting in a rapid decrease in T3. In addition, iopanoic acid inhibits binding of T3 and T4 to cellular receptors. It has been recommended to use in association with anti-thyroid drugs only in certain conditions, mainly in patients with life-threatening thyroid storm (14). Accordingly, we administered iopanoic acid (500 mg twice daily) to the patient, who showed a significant and rapid normalization of T3 levels (table 1) with the simultaneous improvement in clinical condition. A growing number of people worldwide are using “alternative medications”, such as herbal products or dietary supplements, for preventive and alternative therapies. In fact, it has been reported that approximately 25% of Americans who consult their physician about a serious health problem adopt unconventional therapy, but only 70% of these patients tell their physician about it (15, 16). The Dietary Supplement Health and Education Act of 1994 allows these products to be labelled with statements explaining their purported effect on the structure or function of the human body (e.g. alleviation of fatigue) or their role in promoting general well-being (e.g. enhancement of mood or mental activity) (17), and they do not require approval from the Food and Drug Administration or from the European Medicines Agency (15). Alternative medications are considered to have no side effects and also to have no interactions with drugs, thanks to their declared "natural" origin. However, many adverse effects and drug interactions with serious clinical consequences have been reported (15, 18, 19). Very recently, Stub et al. described very elegantly the risk assessment in homeopathic practice. The authors suggest that risk can be divided into direct risk and indirect risk. Direct risk refers to the traditional adverse effects of an intervention; indirect risk is related to adverse effects within the context of a treatment, i.e. depending on the practitioner. Available data suggest that the risk profile of homeopathic remedies in ultramolecular potencies is minor, but there is a potential for indirect risk related to homeopathic practice (20).

We are unable to identify the risk of homeopathic practice in the case in question. However, the high level of iodemia shown in our patient - around a thousand times greater than the normal range and itself associated with fatal outcomes (21) - was caused by long-term homeopathic treatment. This had two consequences: first, iodine load precipitated the development of hyperthyroidism into thyroid storm; and secondly, it prevented us from treating the patient with inorganic iodide.

In conclusion, we described a thyroid storm precipitated by long-term treatment using homeopathic medicine containing iodine.
